# Prevalence trends of depression and anxiety symptoms in adults with cardiovascular diseases and diabetes 1995–2019: *The HUNT studies*, *Norway*

**DOI:** 10.1186/s40359-021-00636-0

**Published:** 2021-08-31

**Authors:** Ivana Bojanić, Erik R. Sund, Hege Sletvold, Ottar Bjerkeset

**Affiliations:** 1 Faculty of Nursing and Health Sciences, Nord University, PB 93, 7601 Levanger, Norway; 2 Department of Public Health and Nursing, Faculty of Medicine and Health Sciences, HUNT Research Centre, Norwegian University of Science and Technology, NTNU, Levanger, Norway; 3 Levanger Hospital, Nord-Trøndelag Hospital Trust, Levanger, Norway; 4 Department of Mental Health, Faculty of Medicine and Health Sciences, Norwegian University of Science and Technology, NTNU, Trondheim, Norway

**Keywords:** Cardiovascular diseases, Diabetes mellitus, Depression symptoms, Anxiety symptoms, Prevalence, Multi-level models

## Abstract

**Background:**

Symptoms of depression and anxiety are common in adults with cardiovascular diseases (CVDs) and diabetes mellitus (DM). The literature on depression and anxiety in CVDs and DM populations is extensive; however, studies examining these relationships over time, directly compared to adults without these conditions, are still lacking. This study aimed to investigate trends in depression and anxiety symptom prevalence over more than 20 years in adults with CVDs and DM compared to the general population.

**Methods:**

We used data from the population-based Trøndelag Health Study (HUNT), Norway, including adults (≥ 20 years) from three waves; the HUNT2 (1995–97; n = 65,228), HUNT3 (2006–08; n = 50,800) and HUNT4 (2017–19; n = 56,042). Depressive and anxiety symptom prevalence was measured independently by the Hospital Anxiety and Depressions scale (HADS) in sex-stratified samples. We analyzed associations of these common psychological symptoms with CVDs and DM over time using multi-level random-effects models, accounting for repeated measurements and individual variation.

**Results:**

Overall, the CVDs groups reported higher levels of depression than those free of CVDs in all waves of the study. Further, depressive and anxiety symptom prevalence in adults with and without CVDs and DM declined from HUNT2 to HUNT4, whereas women reported more anxiety than men. Positive associations of depression and anxiety symptoms with CVDs and DM in HUNT2 declined over time. However, associations of CVDs with depression symptoms remained over time in men. Moreover, in women, DM was associated with increased depression symptom risk in HUNT2 and HUNT4.

**Conclusions:**

Depression and anxiety symptoms are frequent in adults with CVDs. Further, our time trend analysis indicates that anxiety and depression are differentially related to CVDs and DM and sex. This study highlights the importance of awareness and management of psychological symptoms in CVDs and DM populations.

**Supplementary Information:**

The online version contains supplementary material available at 10.1186/s40359-021-00636-0.

## Introduction

Cardiovascular diseases (CVDs) and diabetes mellitus (DM) represent major public health challenges, and their prevalence rates are steadily rising globally. World Health Organization (WHO) estimate that 17.8 million people die from CVDs each year, representing 31% of all global deaths [1]. The global population with DM in 2013 is estimated at 382 million (ages 20 to 79), the number expected to rise to 592 million by 2035 [2]. Simultaneously, a growing prevalence of depression and anxiety has been reported worldwide. In 2017, more than 264 million people of all ages worldwide suffered from depression [3]. The rates varied across studies and countries, yet a systematic review from 2016 concluded that the anxiety prevalence was generally high (3.8–25%) and particularly in women (5.2–8.7%) and individuals from European countries (3.8–10.4%) [4]. Higher prevalence rates and risk of depression and anxiety in women compared to men have been well documented [5, 6]. However, whether depression and anxiety prevalence is increasing over time is still debated [7, 8].

CVDs and DM populations are often affected by higher depression and anxiety symptom load than the general population [9, 10]. Research suggests common biological pathways of CVDs and DM with depression and anxiety, focusing on the autonomic and hypothalamic-pituitary-adrenal (HPA) axis and immuno-inflammatory dysregulation [11, 12]. Depression and anxiety have different clinical manifestations; however, these psychological conditions often overlap [13] and appear together with CVDs and DM [9, 10], further increasing the burden of symptoms in these wide-spread physical illnesses [14].

Worldwide, studies consistently report on increased prevalence rates and risk of depression and anxiety in adults with CVDs compared to people free from these conditions [9, 15–20]. WHO has estimated the prevalence of clinical depression in CVDs populations to range between 3 and 9% worldwide [9] in the last two decades, yet rates between 35 and 46% in China [15] and rates as high as 47% have been reported from Iran [18]. A recent meta-analysis found that of 10,785 acute myocardial patients, approximately one of five were diagnosed with major depression, whereas one of three reported mild to moderate symptoms of depression [19]. A study using data on population-based adults from 17 countries demonstrated a higher odds ratio (OR) for depression (adjusted OR 2.1; 95% CI 1.9–2.5) and anxiety (adjusted OR 1.4; 95% CI 1.0–1.9) in participants with CVDs than those with no such conditions [9]. Likewise, symptoms of depression and anxiety are frequently present in adults with DM [21–23]. Epidemiological evidence suggests that the prevalence of depression is more than three times higher in adults with type 1 DM and almost twice as high in adults with type 2 DM [21]. In line with this, a meta-analysis of longitudinal studies showed that people with DM had an average of 30% higher risk of developing depression than those without [24]. A recent systematic review shows that anxiety disorders and anxiety symptoms are present in 14% and 40% of patients with DM [22]. These findings correspond to a systematic review that reported positive associations between DM and both anxiety disorders (pooled OR 1.20; 95% CI 1.10–1.30) and elevated anxiety symptom levels (pooled OR 1.48; 95% CI 1.02–1.93) [25].

Unfortunately, depression and anxiety symptoms often go undetected and untreated in CVDs and DM populations [26, 27]. In turn, this may contribute to poor treatment outcome of the primary disease [28, 29], reduced quality of life [30, 31] and increased health care costs [32, 33]. Recently, there has, therefore, been a growing interest in CVDs and DM populations' psychological conditions and how to improve the clinical practice of detection and treatment [34, 35]. Despite the increasing literature on depression and anxiety in adults with CVDs and DM, studies examining secular trends in depression and anxiety symptom levels are lacking. Thus, this study aims to investigate the development of depression and anxiety symptom levels in CVDs and DM groups, using population-based data from three waves of the Trøndelag Health Study (HUNT) over more than two decades (HUNT2, HUNT3, and HUNT4). The objectives of this study are: (1) to describe depression and anxiety symptom load in the populations according to CVDs and DM status (2) to investigate time trends of these psychological symptoms over 22-years, and to (3) examine the associations of CVDs and DM with depression and anxiety symptom risk.

## Methods

### Study population

The HUNT Study is a repeated, serial-entry health study of an entire population residing in Nord-Trøndelag county (recently included in the larger Trøndelag County), Norway, carried out in four waves: HUNT1 (1984–86), HUNT2 (1995–97), HUNT3 (2006–08), and HUNT4 (2017–19). The serial-entry participation means that all county inhabitants eligible to participate (aged 19 years and more) were invited every 10 years—regardless of whether they had participated before or not. This study used data from the HUNT2, HUNT3 and HUNT4 waves, where all county adults (aged ≥ 20 years) received questionnaires (Q1) to fill out before the clinical screening test. A second questionnaire (Q2) was distributed to be filled out and returned by mail after clinical examination. Of all invited, the number of respondents whose data material was available to this study was 65,228 (69.5%) in HUNT2, 50,800 (54.1%) in HUNT3, and 56,042 (54%) in HUNT4. The number of participants and the respective participation rates (in %) is per point of data collection, as the number of eligible adults in the county changed over time. Information on the participation of cohorts over time is available on the HUNT official website [36]. The population in HUNT is considered representative of general Norwegian adults [37]. All HUNT participants gave their written consent for research on their data. This study was approved by the Regional Committees for Medical Research and Health Research Ethics in Norway (reference2019/30292/REK Nord) and the Norwegian Centre for Research Data (reference 30292/NSD).

### Data material

**Fig. 1:**
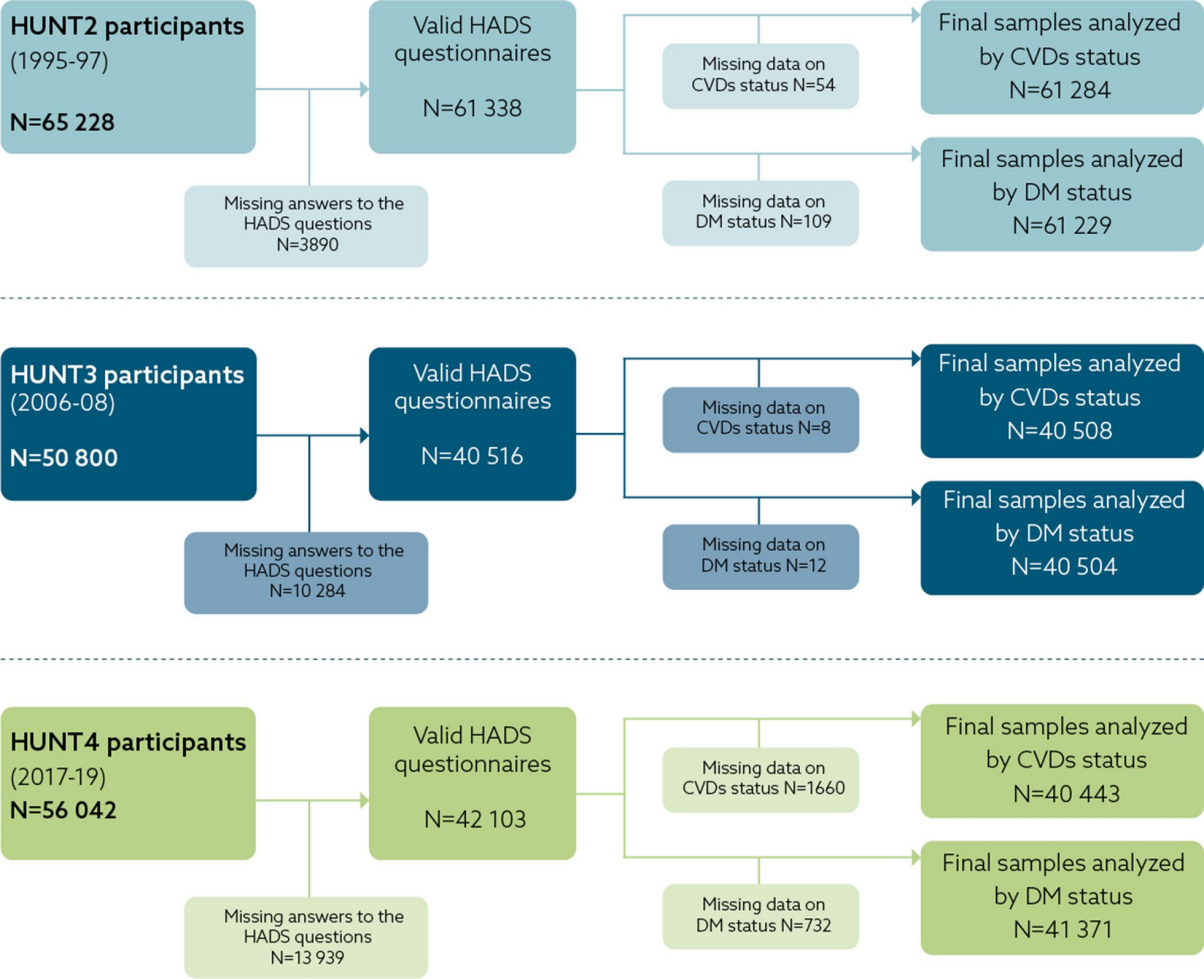
Flow chart showing the selection criteria^a^ for participants. ^a^Hospital Anxiety and Depression Scale (HADS) questionnaires, cardiovascular diseases (CVDs) and diabetes mellitus (DM) Status. CVDs status defined as CVDs or no-CVDs; DM status was defined as DM or no-DM

This study used data from the main questionnaires (Q1 and Q2) covering a wide range of variables on health condition, lifestyle, sociodemographic characteristics, and clinical measurements. The participants yielding valid data on self-reported depression and anxiety questions (outcome) and CVDs and/or DM status (exposure) were eligible, while those with missing values on both exposures were excluded. The study population was analyzed by diseases status (CVDs and DM) independently in sex-stratified samples. Analysis by CVDs status was carried out in 61,284 participants from HUNT2, 40,508 participants from HUNT3 and 40,443 participants from HUNT4, including individuals with and without CVDs. Samples analyzed by DM status were 61,229 participants from HUNT2, 40,504 participants from HUNT3 and 41,371 participants from HUNT4, including individuals with and without DM. Samples of individuals with both CVDs and DM were too small (i.e., 204, 164 and 182 among women and 262, 283 and 385 among men in HUNT2, HUNT3 and HUNT4, respectively) to provide the necessary statistical power and were therefore not analyzed as a separate group. Figure 1 shows the flow chart of the study participants selected for this study.

## Measurements

### Outcome variables: Anxiety and Depression symptoms

The Hospital Anxiety and Depression Scale (HADS) was used to assess symptoms of depression and anxiety. The HADS is a brief self-report questionnaire consisting of 14 items, seven for anxiety subscale (HADS-A) and seven for depression subscale (HADS-D), each scored on a Likert-scale from 0 (no symptoms) to 3 (symptoms maximally present) [38]. For this study, valid ratings of the HADS-D and HADS-A were defined as at least five completed items on both subscales. The score of those who filled in five or six items was based on the sum of completed items multiplied by 7/5 or 7/6, respectively. We assessed anxiety and depression with the categorical approach, using a conventional cut-off threshold of 8 on both the HADS subscales. This cut off value is found to provide optimal sensitivity and specificity (about 0.80) and a good correlation with the case of clinical depression based on DSM-III and ICD-8/9 diagnostic criteria [39]. Additionally, these conventional cut-offs are often used for decision-making purposes, such as rating the severity of depression or the need for treatment [40]. Reliability was examined by ordinal and traditional Cronbach's alpha and performed well on both HADS-A and HADS-D subscales (ordinal alpha was 0.92 and 0.88; Cronbach's alpha was 0.87 and 0.81, respectively) [41]. The HADS-subscales has been confirmed as reliable for detecting symptoms of anxiety and depression independently and describing symptom severity among the CVDs and DM populations [42, 43].

### Exposure variables

CVDs status was measured with questions on the history of heart diseases (myocardial infarct or angina) or stroke (yes/no). Question on heart failure was excluded from HUNT2, and thus, this condition was not used in the definition of CVDs in this study. History of diabetes, including type 1 DM, type 2 DM and other DM types, were criteria for defining DM (yes/no). Missing data on CVDs and DM were defined as an absence of the diseases.

### Other independent variables

Sociodemographic characteristics of the study sample included: sex (classified as women and men), age (mean and age groups < 55, 55–64 and ≥ 65 years) and cohabitation status (living with someone vs living alone). The HUNT3 database lacks direct data describing socioeconomic status (e.g., education level). Therefore, we used the lifestyle variable "current smoking" (yes/no) as an indicator of socioeconomic status in multivariate analysis. Other lifestyle measurements included monthly alcohol consumption (no or low drinking versus moderate to frequent) and physical activity (inactive versus active). Alcohol consumption in HUNT2 was described numerically (i.e., times drinking per month) whereas in HUNT3 and HUNT4 with categories. In this study, never or ≤ one time per week was defined as no or low drinking, while drinking two-three times or ≥ four times per week was moderate to frequent drinking. In HUNT, leisure-time physical activity was measured by questions about light (i.e., no sweating or heavy breathing) and hard (i.e., sweating, and heavy breathing) physical activity per week. We defined the respondents with no physical activity or less than one time per week as not physically active, while those with more than one time per week of hard/let physical activity were physically active. Of clinical anthropometric measurements, we used Body mass index (BMI) as a general indicator of overweight and obesity, which are significant risk factors for cardio-metabolic diseases [44], depression [45] and anxiety [46]. Body mass index (BMI) included two categories: underweight to normal (< 25 kg/m^2^) and overweight to obese (≥ 25 kg/m^2^), defined according to WHO BMI cut-off for overweight and obesity classification [47].

### Statistical analysis

The prevalence of self-reported anxiety and depression symptoms was evaluated using cross-sectional data from three HUNT surveys performed with an 11-years' interval. Descriptive statistics regarding baseline characteristics included frequencies and percentages. The study population's characteristics stratified by sex are presented for the sample with a report on CVDs (Table 1) and DM status (Table 2) separately. The groups with positive disease status in Tables 1 and 2 are bolded. Prevalence of depression and anxiety was described separately by disease status and across sexes. Estimates were age-standardized (using the age categories < 55, 55–64 and ≥ 65 years) by the direct standardization using the age distribution of participants attending the screening in HUNT3 as the standard population. The associations of self-reported depression and anxiety with CVDs and DM were analyzed using multi-level logistic models. Multi-level models were specified to account for repeated measurements on the same participants (i.e., non-independent observations). To derive risk ratios (RR) and risk differences (RD), we used predictions from the multi-level models. Specifically, the RR was formed as the ratio between the mean predicted probability, whereas RD was the difference in mean predicted probability. We calculated RR and RD for three specific ages, 40, 60 and 80. We present findings for age 60 in the results section and age 40 and 80 in the supplementary information (see Additional files 1 and 2 for CVDs and DM analysis, respectively). Associations of diseases status (i.e., CVDs and DM) and self-reported depression and anxiety are reported with 95 per cent confidence intervals (95% CI). All statistical models were sex-stratified. The models considered first age adjustment (age and age squared), followed by the inclusion of other sociodemographic variables (i.e., smoking and cohabitation) and finally, lifestyle measurements (i.e., alcohol consumption, physical activity and BMI). First we adjusted for BMI using categorical approach with cut-off at 25 kg/m^2^. Second, we used BMI as a continuous variable with restricted cubic splines and tested for possible non-linear associations between the continuous change of BMI and the outcome (anxiety and depression symptoms) at four prespecified locations according to percentiles of BMI distribution (i.e., 5th, 25th, 75th and 95th) that correspond to different BMI values (i.e., 20.7 kg/m^2^, 24.1 kg/m^2^, 26.3 kg/m^2^, 28.9 kg/m^2^, and 34.9 kg/m^2^, respectively).Statistically significant associations of self-reported depression and anxiety with CVDs (Table 3) and DM (Table 4) are highlighted in bold. The statistical software STATA® (version 16) was used in the analysis.

**Table 1:** The characteristics of HUNT2, HUNT3 and HUNT4 participants according to CVDs status stratified by sex

Total n (%)	HUNT2 (1995–98) n = 61,284				HUNT3 (2006–08) n = 40,508				HUNT4 (2017–19) n = 40,443			
	CVDs n = 4484 (7.3)		No-CVDs n = 56,800 (92.7)		CVDs n = 3472 (8.6)		No-CVDs n = 37,036 (91.4)		CVDs n = 3531 (8.7)		No-CVDs n = 36,912 (92.3)	
	Women n = 1746 (38.9)	Men n = 2738 (61.1)	Women n = 30,552 (53.8)	Men n = 26,248 (46.2)	Women n = 1324 (38.1)	Men n = 2148 (61.9)	Women n = 21,359 (57.7)	Men n = 15,677 (42.3)	Women n = 1354 (38.4)	Men n = 2177 (61.6)	Women n = 21,690 (58.8)	Men n = 15,222 (41.2)
Variables												
Depression^a^												
No	**1344 (77.0)**	**2181 (79.7)**	27,538 (90.1)	23,560 (89.8)	**1092 (82.5)**	**1805 (84.0)**	19,583 (91.7)	14,122 (90.1)	**1187 (87.7)**	**1854 (85.2)**	19,806 (91.3)	13,773 (90.5)
Yes	**402 (23.0)**	**557 (20.3)**	3014 (9.9)	2688 (10.2)	**232 (17.5)**	**343 (16.0)**	1776 (8.3)	1555 (9.9)	**167 (12.3)**	**323 (14.8)**	1884 (8.7)	1449 (9.5)
Anxiety^b^												
No	**1231 (70.5)**	**2228 (81.4)**	23,196 (75.9)	21,701 (82.7)	**1044 (78.8)**	**1914 (89.1)**	17,695 (82.8)	14,068 (89.7)	**1077 (79.5)**	**1929 (88.6)**	17,120 (78.9)	13,190 (86.7)
Yes	**515 (29.5)**	**510 (18.6)**	7356 (24.1)	4547 (17.3)	**280 (21.2)**	**234 (10.9)**	3664 (17.2)	1609 (10.3)	**277 (20.5)**	**248 (11.4)**	4570 (21.1)	2032 (13.3)
Age (years)												
Mean (Sd)^c^	**71.6 (10.8)**	**68.0 (15.6)**	47.6 (16.3)	47.1 (10.7)	**70.4 (11.9)**	**68.8 (10.8)**	52.5 (15.6)	53.5 (14.6)	**70.5 (14.0)**	**70.8 (10.7)**	53.6 (16.8)	55.2 (16.1)
<55	**137 (7.9)**	**353 (12.9)**	21,014 (68.8)	18,454 (70.3)	**137 (10.4)**	**222 (10.3)**	11,686 (54.7)	8096 (51.7)	**174 (12.9)**	**167 (7.7)**	11,018 (50.8)	7036 (46.2)
55–64	**257 (14.7)**	**566 (20.7)**	4162 (13.6)	3649 (13.9)	**257 (19.4)**	**525 (24.4)**	4903 (23.0)	4097 (26.1)	**197 (14.5)**	**355 (16.3)**	4574 (21.1)	3442 (22.6)
≥65	**1352 (77.4)**	**1819 (66.4)**	5376 (17.6)	4145 (15.8)	**930 (70.2)**	**1401 (65.3)**	4770 (22.3)	3484 (22.2)	**983 (72.6)**	**1655 (76.0)**	6098 (28.1)	4744 (31.2)
Cohabitation												
Living with someone	**780 (44.7)**	**1889 (69.0)**	21,610 (70.7)	17,237 (65.7)	**778 (58.8)**	**1766 (82.2)**	17,147 (80.3)	13,064 (83.3)	**785 (58.0)**	**1737 (79.8)**	16,810 (77.5)	12,466 (81.9)
Living alone	**966 (55.3)**	**849 (31.0)**	8942 (29.3)	9011 (34.3)	**546 (41.2)**	**382 (17.8)**	4212 (19.7)	2613 (16.7)	**569 (42.0)**	**440 (20.2)**	4880 (22.5)	2756 (18.1)
Current smocking												
No	**1361 (78.0)**	**2034 (74.3)**	20,755 (67.9)	18,380 (70.1)	**999 (75.5)**	**1650 (76.8)**	15,673 (73.4)	11,947 (76.2)	**1182 (87.3)**	**1978 (90.8)**	19,408 (89.5)	13,985 (91.9)
Yes	**314 (18.0)**	**661 (24.1)**	9226 (30.2)	7544 (28.7)	**257 (19.4)**	**436 (20.3)**	5135 (24.0)	3363 (21.5)	**161 (11.9)**	**189 (8.7)**	2216 (10.2)	1203 (7.9)
Missing	**71 (4.0)**	**43 (1.6)**	571 (1.9)	324 (1.2)	**68 (5.1)**	**62 (2.9)**	551 (2.6)	367 (2.3)	**11 (0.8)**	**10 (0.5)**	66 (0.3)	34 (0.2)
Physical activity												
Inactive^d^	**252 (14.4)**	**212 (7.8)**	1511 (5.0)	1493 (5.7)	**135 (10.2)**	**189 (8.8)**	730 (3.4)	871 (5.6)	**130 (9.6)**	**139 (6.4)**	631 (2.9)	628 (4.1)
Active^e^	**948 (54.3)**	**2057 (75.1)**	25,941 (84.9)	22,924 (87.3)	**1130 (85.3)**	**1911 (89.0)**	20,293 (95.0)	14,602 (93.1)	**1177 (86.9)**	**1986 (91.2)**	20,761 (95.7)	14,414 (94.7)
Missing	**546 (31.3)**	**469 (17.1)**	3100 (10.1)	1831 (7.0)	**59 (4.5)**	**48 (2.2)**	336 (1.6)	204 (1.3)	**47 (3.5)**	**52 (2.4)**	298 (1.4)	180 (1.2)
Alcohol consumption												
No or low^f^	**1522 (87.2)**	**2127 (77.7)**	25,814 (84.5)	20,008 (76.2)	**977 (73.8)**	**1286 (59.9)**	14,202 (66.5)	8329 (53.2)	**1159 (85.6)**	**1641 (75.4)**	17,946 (82.7)	11,014 (72.4)
Moderate to frequent^g^	**44 (2.5)**	**311 (11.4)**	2275 (7.4)	4615 (17.6)	**257 (19.4)**	**802 (37.3)**	6570 (30.8)	7092 (45.2)	**157 (11.6)**	**499 (22.9)**	3516 (16.2)	4074 (26.7)
Missing	**180 (10.3)**	**300 (10.9)**	2463 (8.1)	1625 (6.2)	**90 (6.8)**	**60 (2.8)**	587 (2.7)	256 (1.6)	**38 (2.8)**	**37 (1.7)**	228 (1.1)	134 (0.9)
BMI^h^ (kg/m^2^)												
Underweight to normal	**471 (27.0)**	**740 (27.0)**	13,942 (45.7)	9405 (35.8)	**333 (25.2)**	**447 (20.8)**	8317 (38.9)	3915 (25.0)	**396 (29.3)**	**446 (20.5)**	8522 (39.3)	4141 (27.2)
Overweight to obese	**1178 (67.5)**	**1939 (70.8)**	16,323 (53.4)	16,685 (63.6)	**974 (73.6)**	**1682 (78.3)**	12,978 (60.8)	11,725 (74.8)	**927 (68.5)**	**1689 (77.6)**	13,052 (60.2)	11,007 (72.3)
Missing	**97 (5.5)**	**59 (2.2)**	287 (0.9)	158 (0.6)	**17 (1.2)**	**19 (0.9)**	64 (0.3)	37 (0.2)	**31 (2.2)**	**42 (1.9)**	116 (0.5)	74 (0.5)

CVDs, Cardiovascular diseases; HUNT, The Trøndelag Health Study

^a^Depression symptoms defined by HADS-D ≥ 8, HADS-D Hospital Anxiety and Depression-subscale Depression

^b^Anxiety symptoms defined by HADS-A ≥ 8, HADS-A Hospital Anxiety and Depression-subscale Anxiety

^c^Sd = standard deviation

^d^Inactive = never or no light/hard physical activity per week

^e^Active = less than once or more light/hard physical activity per week

Light physical activity (no sweating or heavy breathing) vs hard physical activity

^f^No or low drinking = Never or ≤ 1 time/week

^g^Moderate (2–3 times/week) to frequent (≥ 4 times/week)

^h^BMI = Body mass index; underweight to normal: BMI < 25 kg/m^2^; overweight to obese: BMI ≥ 25 kg/m^2^

**Table 2:** The characteristic of HUNT2, HUNT3 and HUNT4 study participants according to DM stratified by sex

Total n (%)	HUNT2 (1995–98) n = 61,229				HUNT3 (2006–08) n = 40,504				HUNT4 (2017–19) n = 41,371			
	DM n = 1707 (2.8)		No-DM n = 59,522 (97.2)		DM n = 1872 (4.6)		No-DM n = 38,632 (95.4)		DM n = 2569 (6.2)		No-DM n = 38,802 (93.8)	
	Women n = 847 (49.6)	Men n = 860 (50.4)	Women n = 31,435 (52.8)	Men n = 28,087 (47.2)	Women n = 904 (48.3)	Men n = 968 (51.7)	Women n = 21,776 (56.4)	Men n = 16,856 (43.6)	Women n = 1217 (47.4)	Men n = 1352 (52.6)	Women n = 22,436 (57.8)	Men n = 16,366 (42.2)
Variables												
Depression^a^												
No	**692 (81.7)**	**710 (82.6)**	28,182 (89.7)	25,004 (89.0)	**791 (87.5)**	**836 (86.4)**	19,881 (91.3)	15,091 (89.5)	**1052 (86.4)**	**1171 (86.6)**	20,469 (91.2)	14,713 (89.9)
Yes	**155 (18.3)**	**150 (17.4)**	3253 (10.3)	3083 (11.0)	**113 (12.5)**	**132 (13.6)**	1895 (8.7)	1765 (10.5)	**165 (13.6)**	**181 (13.4)**	1967 (8.8)	1653 (10.1)
Anxiety^b^												
No	**631 (74.5)**	**702 (81.6)**	23,791 (75.7)	23,192 (82.6)	**748 (82.7)**	**866 (89.5)**	17,988 (82.6)	15,115 (89.7)	**936 (76.9)**	**1181 (87.4)**	17,702 (78.9)	14,184 (86.7)
Yes	**216 (25.5)**	**158 (18.4)**	7644 (24.3)	4895 (17.4)	**156 (17.3)**	**102 (10.5)**	3788 (17.4)	1741 (10.3)	**281 (23.1)**	**171 (12.6)**	4734 (21.1)	2182 (13.3)
Age (years)												
Mean (Sd)^c^	**66.1 (14.9)**	**63.6 (14.5)**	48.4 (16.8)	48.6 (16.3)	**64.7 (13.2)**	**64..6 (11.3)**	53.1 (15.9)	54.8 (15.0)	**65.0 (14.6)**	**67.5 (11.4)**	52.3 (17.1)	56.4 (16.5)
<55	**194 (22.9)**	**232 (27.0)**	20,952 (66.6)	18,564 (66.1)	**189 (20.9)**	**174 (18.0)**	11,633 (53.4)	8144 (48.3)	**270 (22.2)**	**183 (13.5)**	11,075 (49.4)	7105 (43.4)
55–64	**123 (14.5)**	**157 (18.3)**	4291 (13.7)	4048 (14.4)	**244 (27.0)**	**309 (31.9)**	4916 (22.6)	4312 (25.6)	**242 (19.9)**	**292 (21.6)**	4634 (20.7)	3566 (21.8)
≥65	**530 (62.6)**	**471 (54.7)**	6192 (19.7)	5475 (19.5)	**471 (52.1)**	**485 (50.1)**	5227 (24.0)	4400 (26.1)	**705 (57.9)**	**877 (64.9)**	6727 (29.9)	5695 (34.8)
Cohabitation												
Living with someone	**427 (50.4)**	**565 (65.7)**	21,950 (69.8)	18,536 (66.0)	**618 (68.4)**	**798 (82.4)**	17,306 (79.5)	14,031 (83.2)	**804 (66.1)**	**1070 (79.1)**	17,204 (76.7)	13,351 (81.6)
Living alone	**420 (49.6)**	**295 (34.3)**	9485 (30.2)	9551 (34.0)	**286 (31.6)**	**170 (17.6)**	4470 (20.5)	2825 (16.8)	**413 (33.9)**	**282 (20.9)**	5232 (23.3)	3015 (18.4)
Current smoking												
No	**695 (82.1)**	**647 (75.3)**	21,408 (68.1)	19,739 (70.3)	**696 (77.0)**	**768 (79.3)**	15,974 (73.4)	12,827 (76.1)	**1070 (87.9)**	**1241 (91.8)**	20,024 (89.3)	14,997 (91.6)
Yes	**118 (13.9)**	**192 (22.3)**	9419 (30.0)	8003 (28.5)	**167 (18.5)**	**180 (18.6)**	5224 (24.0)	3620 (21.5)	**133 (10.9)**	**105 (7.8)**	2321 (10.3)	1318 (8.1)
Missing	**34 (4.0)**	**21 (2.4)**	608 (1.9)	345 (1.2)	**41 (4.5)**	**20 (2.1)**	578 (2.6)	409 (2.4)	**14 (1.2)**	**6 (0.4)**	91 (0.4)	51 (0.3)
Physical activity												
Inactive^d^	**102 (12.0)**	**66 (7.7)**	1660 (5.3)	1639 (5.8)	**76 (8.4)**	**85 (8.8)**	788 (3.6)	976 (5.8)	**87 (7.2)**	**99 (7.3)**	701 (3.1)	683 (4.2)
Active^e^	**504 (59.5)**	**656 (76.3)**	26,374 (83.9)	24,291 (86.5)	**801 (88.6)**	**868 (89.7)**	20,620 (94.7)	15,643 (92.8)	**1099 (90.3)**	**1226 (90.7)**	21,387 (95.3)	15,456 (94.4)
Missing	**241 (28.5)**	**138 (16.0)**	3401 (10.8)	2157 (7.7)	**27 (3.0)**	**15 (1.5)**	368 (1.7)	237 (1.4)	**31 (2.5)**	**27 (2.0)**	348 (1.6)	227 (1.4)
Alcohol consumption												
No or low^f^	**746 (88.1)**	**666 (77.5)**	26,572 (84.5)	21,435 (76.3)	**719 (79.5)**	**627 (64.8)**	14,458 (66.4)	8988 (53.3)	**1089 (89.5)**	**1091 (80.7)**	18,515 (82.5)	11,809 (72.2)
Moderate to frequent^g^	**26 (3.1)**	**106 (12.3)**	2293 (7.3)	4816 (17.2)	**146 (16.2)**	**323 (33.4)**	6680 (30.7)	7570 (44.9)	**97 (8.0)**	**241 (17.8)**	3649 (16.3)	4385 (26.8)
Missing	**75 (8.8)**	**88 (10.2)**	2570 (8.2)	1836 (6.5)	**39 (4.3)**	**18 (1.8)**	638 (2.9)	298 (1.8)	**31 (2.5)**	**20 (1.5)**	272 (1.2)	172 (1.0)
BMI^h^ (kg/m^2^)												
Underweight to normal	**154 (18.2)**	**198 (23.0)**	14,251 (45.3)	9945 (35.4)	**140 (15.5)**	**139 (14.4)**	8508 (39.1)	4222 (25.1)	**214 (17.6)**	**200 (14.8)**	8907 (39.7)	4454 (27.2)
Overweight to obese	**651 (76.9)**	**646 (75.1)**	16,841 (53.6)	17,952 (63.9)	**751 (83.1)**	**819 (84.6)**	13,200 (60.6)	12,588 (74.7)	**989 (81.3)**	**1131 (83.7)**	13,397 (59.7)	11,817 (72.2)
Missing	**42 (4.9)**	**16 (1.9)**	343 (1.1)	190 (0.7)	**13 (1.4)**	**10 (1.0)**	68 (0.3)	46 (0.2)	**14 (1.1)**	**21 (1.5)**	132 (0.6)	95 (0.6)

DM, Diabetes mellitus; HUNT, The Trøndelag Health Study

^a^Depression symptoms defined by HADS-D ≥ 8, HADS-D Hospital Anxiety and Depression-subscale Depression,

^b^Anxiety symptoms defined by HADS-A ≥ , HADS-A Hospital Anxiety and Depression-subscale Anxiety

^c^Sd = standard deviation

^d^Inactive = never or no light/hard physical activity per week

^e^Active = less than once or more light/hard physical activity per week

Light physical activity (no sweating or heavy breathing) vs hard physical activity

^f^No or low drinking = Never or ≤ 1 time/week

^g^Moderate (2–3 times/week) to frequent (≥ 4 times/week)

^h^BMI = Body mass index; underweight to normal: BMI < 25 kg/m^2^; overweight to obese: BMI ≥ 25 kg/m^2^

## Results

### Study population characteristics

Table 1 shows the HUNT2, HUNT3 and HUNT4 study participants' characteristics within sex-stratified samples according to CVDs status. Overall, the prevalence of CVDs was relatively stable from HUNT2 to HUNT4 (range from 7.3 to 8.7%) and higher in women than men. CVDs groups consistently reported higher rates of depression than the no-CVDs group across age, sexes and study waves, yet for anxiety, this pattern was only observed in women and limited to the first two waves of the study. In contrast, DM prevalence consistently increased from HUNT2 to HUNT4 (range from 2.8 to 6.2%), with rates slightly higher in men than women, whereas differences in depression and anxiety symptom load between DM and no-DM groups were less prominent (Table 2). Participants reporting disease (i.e., CVDs or DM) were often 65 years and older, non-smokers, physically active, reported no to low monthly alcohol consumption and were more often overweight or obese.

**Fig. 2:**
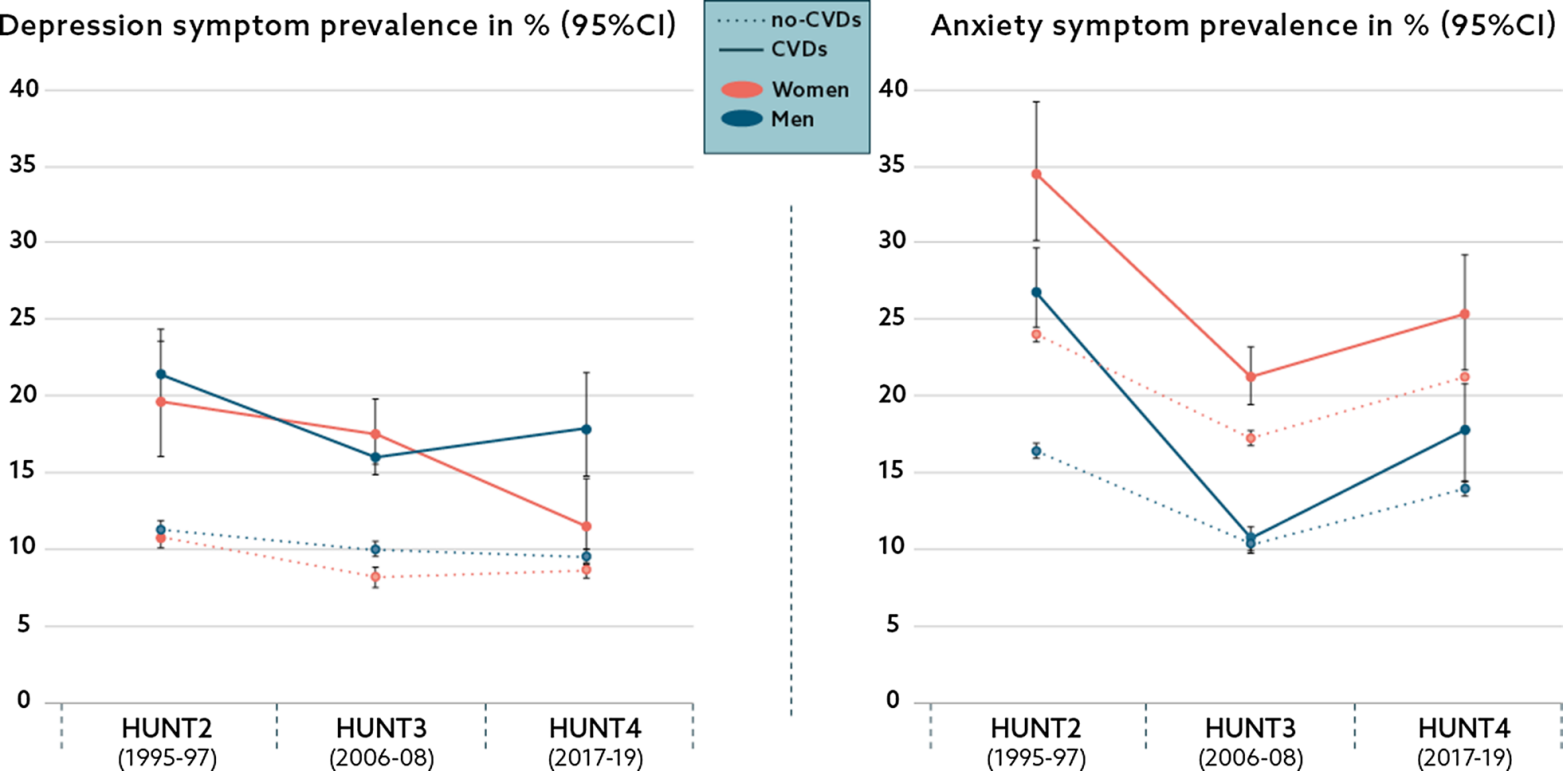
Trends in depression and anxiety symptom prevalence^a^ according to cardiovascular diseases (CVDs) status, the HUNT 1995–2019. ^a^Age-standardized using the age distribution of participants attending a screening in HUNT3 as the standard population. HUNT, The Trøndelag Health Study

Figure 2 shows the age-standardized anxiety and depression symptom prevalence of participants with CVDs compared to the no-CVDs group for the study period 1995–2019. Within CVDs groups, the symptom of depression decreased consistently in women whereas initially declined and subsequently increased in men, the trends resulting in an overall decrease in both sexes over the total study period (from HUNT2 to HUNT4). On the other hand, the trend in depressive symptom prevalence in groups with no CVDs was stable. Anxiety symptom prevalence declined in the first (1995–2008) and increased in the last study period (2008–2019) across all study groups and sexes, resulting in an overall decrease in participants with CVDs and relative stability in groups without this condition. Women reported higher anxiety scores than men, a trend observed across all study groups and periods.

**Fig. 3:**
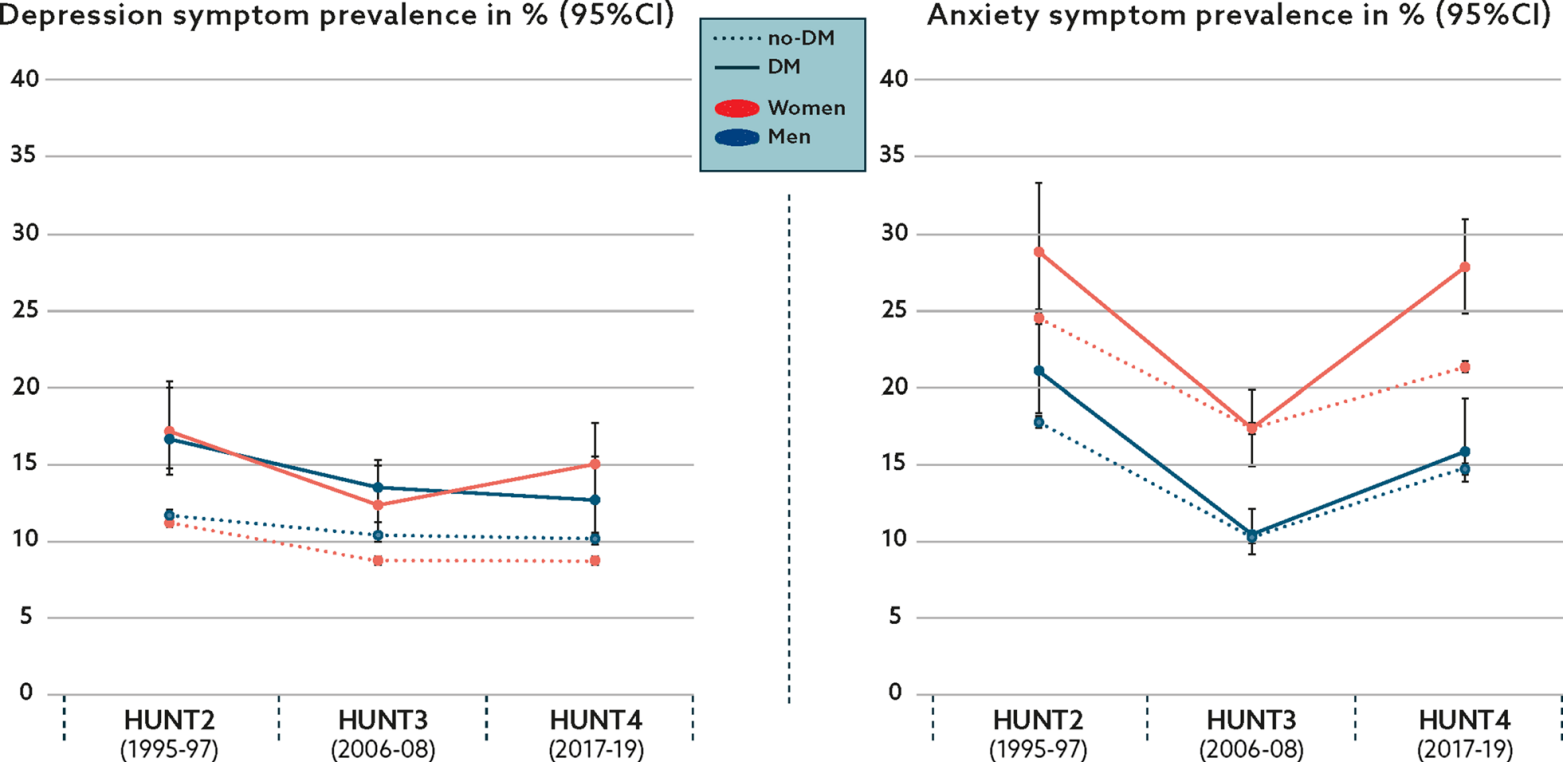
Trends in depression and anxiety symptom prevalence^a^ according to diabetes mellitus (DM) status, the HUNT 1995–2019. ^a^Age-standardized using the age distribution of participants attending a screening in HUNT3 as the standard population. HUNT, The Trøndelag Health Study

Similarly, Fig. 3 shows the age-standardized prevalence of symptoms of anxiety and depression in participants according to DM status. Within DM groups of both sexes, depressive symptom prevalence showed an overall decline in the first period yet increased slightly in women and remained largely unchanged in men throughout the study period. Such trend resulted in an overall depressive symptom decrease in men with DM and relative symptom stability in women with this condition. From HUNT2 to HUNT3, anxiety symptom prevalence declined across all study groups and sexes and subsequently increased again in HUNT4, so that anxiety prevalence rates in DM groups remained largely unchanged in women and declined in men. As in CVDs analyses, depression symptom prevalence remained relatively stable in no-DM participants of both sexes, whereas anxiety symptoms increased in the first and declined in the last period.

### Associations of cardiovascular diseases and diabetes with depression and anxiety symptoms at age 60

Table 3 shows associations of CVDs with symptoms of depression and anxiety at age 60 years for men and women in HUNT2, HUNT3 and HUNT4. Overall, the risk differences between individuals with and without CVDs declined over time for both sexes yet remained statistically significant in men. Among women, RD for depression decreased from 0.08 (95% CI 0.07–0.1) in HUNT2 to 0.05 (95% CI 0.04–0.07) in HUNT3. In HUNT4, there was no statistical evidence for any difference between those with and without CVDs on either depression or anxiety symptoms among women. Men with CVDs in HUNT4 had a 26% higher risk for symptoms of depression than males with no CVDs, with an absolute RD of 0.03 (95% CI 0.01–0.04). In contrast, there was no statistical evidence for any difference between CVDs groups and anxiety symptoms among men in HUNT4.

**Table 3:** Associations of CVDs with depression and anxiety symptoms in HUNT2 (1995–97), HUNT3 (2006–08) and HUNT4 (2017–19), multi-level logistic analysis

	HUNT2 (1995–97)	HUNT3 (2006–08)	HUNT4 (2017–19)	HUNT2 (1995–97)	HUNT3 (2006–08)	HUNT4 (2017–19)
	RR (95% CI)	RR (95% CI)	RR (95% CI)	RD (95% CI)	RD (95% CI)	RD (95% CI)
Depression						
Women n = 45,726	**1.72 (1.55–1.88)**	**1.57 (1.37–1.76)**	1.10 (0.94–1.26)	**0.08 (0.07–0.10)**	**0.05 (0.04–0.07)**	0.01 (− 0.01 to 0.03)
Men n = 39,012	**1.45 (1.33–1.57)**	**1.25 (1.12–1.38)**	**1.26 (1.12–1.39)**	**0.06 (0.04–0.07)**	**0.03 (0.01–0.04)**	**0.03 (0.01 to 0.04)**
Anxiety						
Women n = 45,095	**1.39 (1.28–1.49)**	**1.28 (1.15–1.40)**	1.03 (0.93–1.14)	**0.09 (0.07–0.11)**	**0.05 (0.03–0.07)**	0.01 (− 0.01 to 0.03)
Men n = 38,754	**1.37 (1.25–1.48)**	**1.28 (1.12–1.43)**	1.07 (0.95–1.19)	**0.06 (0.04–0.08)**	**0.03 (0.01–0.04)**	0.01 (− 0.01 to 0.03)

Adjusted for age and age squared. Risk Ratio (RR) and Risk Difference (RD) between individuals reporting CVDs and no-CVDs (ref.) at age 60

CVDs, Cardiovascular diseases; HUNT, The Trøndelag Health Study; RR, Risk ratio; RD, Risk difference; CI, Confidence Interval

Table 4 shows that among adults at the age of 60 with DM in HUNT2, the risk for depression and anxiety symptoms above cut-off levels was raised by 36% compared to no-DM groups as a reference, with an RD of 0.04 (95% CI 0.02–0.07). There was no difference between DM and anxiety or depression risks in either sex in HUNT3, whereas 11 years later (HUNT4), DM was associated with a 24% increased risk for depression and 13% increased risk for anxiety in women but not in men. Further adjustment for sociodemographic and lifestyle variables yielded minimal changes in risk estimates in both CVDs and DM analysis (Tables 3, 4), and thus, these variables were not included in the final model.

**Table 4:** Associations of DM with depression and anxiety symptoms in HUNT2 (1995–97), HUNT3 (2006–08) and HUNT4 (2017–19), multi-level logistic analysis

	HUNT2 (1995–97)	HUNT3 (2006–08)	HUNT4 (2017–19)	HUNT2 (1995–97)	HUNT3 (2006–08)	HUNT4 (2017–19)
	RR (95% CI)	RR (95% CI)	RR (95% CI)	RD (95% CI)	RD (95% CI)	RD (95% CI)
Depression						
Women n = 45,844	**1.36 (1.17–1.56)**	1.18 (0.98–1.38)	**1.24 (1.06–1.42)**	**0.04 (0.02–0.07)**	0.02 (− 0.00 to 0.04)	0.02 (0.01 to 0.04)
Men n = 39,095	**1.22 (1.04–1.40)**	1.07 (0.90–1.24)	1.09 (0.94–1.23)	**0.03 (0.01–0.05)**	0.01 (− 0.01 to 0.03)	0.01 (− 0.01 to 0.03)
Anxiety						
Women n = 45,219	1.12 (1.00–1.25)	1.05 (0.91–1.19)	**1.13 (1.02–1.24)**	0.03(− 0.00 to 0.06)	0.01 (− 0.02 to 0.03)	**0.03 (0.01–0.05)**
Men n = 38,838	**1.21 (1.04–1.38)**	1.13 (0.93–1.33)	1.12 (0.97–1.26)	**0.03 (0.01–0.06)**	0.01 (− 0.01 to 0.03)	0.02 (− 0.00 to 0.04)

Adjusted for age and age squared. risk ratio (RR) and risk difference (RD) between individuals reporting DM and no-DM (ref.) at age 60

DM, diabetes mellitus; HUNT, The Trøndelag Health Study; RR, risk difference; RD, risk ratio; CI, confidence interval

## Discussion

Findings from three waves (1995–2019) of this population-based study of more than 140,000 adults showed higher depression and anxiety symptom prevalence in groups with CVDs and DM than no-disease groups, and differences were generally more pronounced in CVDs than DM. Overall, there was a general decline in depression symptom prevalence in the same period for all study groups. Anxiety symptom prevalence decreased initially and increased in the last decade across study groups and sexes; still, there was an overall symptom reduction in participants with and without CVDs or DM. These trends are not in keeping with a meta-analysis that suggested no change in the global prevalence of depression and anxiety in the general populations in 21 world regions between 1990 and 2010 [7]. Nevertheless, our results partially reflect patterns of depression and anxiety prevalence in other Scandinavian countries [48–50] and confirm existing evidence of the higher prevalence of depressive and anxiety symptoms in populations with CVDs or DM than in the general adults [9, 10].

### Prevalence of depression and anxiety symptoms according to cardiovascular disease and diabetes status

Depression and anxiety symptoms and disorders often overlap with CVDs and DM [17, 24, 25, 51] and are more frequent in people with these wide-spread physical conditions than in the general population [9, 10]. Largely in line with our findings, worldwide population-based survey data from 17 countries, showed that the prevalence of clinically diagnosed depression and anxiety is generally higher in CVDs and DM populations than those with no such conditions, consistently across countries, sexes, and age [9, 10]. However, although not directly comparable, the depressive symptom prevalence in CVDs groups for the period 2006–08 (HUNT3) in our data was closer to the prevalence reported for the corresponding period in the recent US studies—ranging from 15.8 to 18.3% [52, 53], than the pooled depressive symptom prevalence in community-dwelling adults with CVDs in China and Iran ranging from 35 to 47% [15, 18]. Thus, our findings broadly agree with the evidence on the prevalence of depression (i.e., self-reported or clinically diagnosed) in the people with CVDs and the general public to be lower in Western countries than in non-Western world region [54, 55].

### Secular trends in the prevalence of depression and anxiety symptoms

Although it is well established that depression and anxiety prevalence is generally higher in people with chronic medical conditions than those without, findings on time changes in depression and anxiety symptom prevalence in the general population and groups with CVDs or DM have been inconsistent. Epidemiological studies from the last two decades have observed an overall increase in depression and anxiety prevalence in the general population [48, 56, 57] and CVDs and DM populations [10, 57, 58]. In contrast, other studies report that these mental conditions are on the rise in general adult populations [7, 59, 60] and populations with CVDs and DM [23, 53, 61].

In contrast to our findings, studies from the USA showed that depressive symptom prevalence increased in the general population from 2005 to 2016 [57], while no change was found in community-dwelling adults with heart disease (aged 20–80 years) in the same period [53]. Moreover, our prevalence rates of non-disease groups align with the literature review and meta-analysis of studies on the global prevalence of depression and anxiety symptoms, revealing relative stable rates from 1990 through 2005 to 2010 [7]. However, the overall decline of depression symptoms across CVDs/DM groups in this study corresponds to a general reduction in the pooled global prevalence of depression symptoms and disorders observed in various outpatient groups from 1995 to 2010, from 83 cross-sectional studies mainly from Europe, Asia and North America [54]. This change was partly explained by improved treatment and awareness of these psychological conditions [54]. Similarly, the decline in depressive symptom rates was observed in a population-based sample of Mexican adults with DM (aged ≥ 50 years) from 2001 to 2015 [23].

A decrease in anxiety symptom prevalence from 1995 to 2008 in our data is not in keeping with a meta-analysis that relied on data from 44 countries and concluded no change in the global prevalence of anxiety in adults (clinically diagnosed or self-reported) for the period 1990–2010 [8]. On the other hand, Swedish findings from 1980 to 2005 showed a general increase in self-reported anxiety rates among adults aged 16–63 years [48]. Nevertheless, the same study also observed a decline in anxiety symptom prevalence in the oldest female groups (aged 64–71 years), in line with trends observed in our data. Similarly, another population-based Swedish study that examined time trends in self-reported anxiety from 1997 to 2006 reported an increase in participants aged ≤ 24 years, whereas a decrease or stable estimates in the other adult groups (25 years and more) from 2001 and onwards [49]. A study of national representative Dutch adults (aged 18–64 years) observed no change in the prevalence of clinically diagnosed anxiety and depression from 1995 to 2009 [50].

Variations in instruments and criteria, sampling, study location/country, and characteristics of underlying populations make it difficult to directly compare and interpret study findings [62, 63]. In sum, changes in depression and anxiety symptom prevalence for the period 1995–2019 (HUNT2 to HUNT4) within a national representative sample of Norwegian adults are in keeping with comparable studies reporting on depression and anxiety symptom trends in the same age groups in other Scandinavian countries [48, 50]. Moreover, anxiety symptom rates for the period 2006–19 (HUNT3 to HUNT4) showed a marked increase across all study groups and in both sexes. The increase in anxiety symptoms has been linked to the global rise in psychological stressors such as work-life stress [64], urbanization [65] and social media use [66] observed in past decades. However, understanding the extent to which our findings on increasing anxiety prevalence reflect the growing trend in stress-related risk factors for anxiety, particularly within specific subgroups, requires further investigation. On the other hand, the overall reduction in symptoms of depression and anxiety in our study over two decades may, in addition to the above-mentioned cohort effect, to some degree be a result of altered lifestyle behaviour (i.e., non-smoking, physical activity and no-low alcohol use) of study participants becoming "healthier" from HUNT2 to HUNT3, particularly in those with a diagnosis of CVDs and DM, which in turn could have contributed to improved mental health outcomes [67–69]. However, the overall decrease or relative stability of mental health symptoms in our data may reflect an overall improved public recognition of common mental conditions, particularly in groups with wide-spread physical conditions or increased awareness of people with such diseases to seek mental health help [60, 70].

Importantly, the diagnostic criteria for several physical illnesses have changed around the time of HUNT2 [71]. These changes lowered the thresholds for CVDs and DM diagnosis, contributing to higher prevalence and a "healthier" population with these conditions [72, 73]. This change might, at least partly, contribute to the general decline of the existing depression symptom burden across the three HUNT surveys and the drop in anxiety symptoms from HUNT 2 to HUNT3. Nevertheless, these changes have most likely affected CVDs and DM populations similarly in most of the world—both in terms of physical and mental symptom burden.

Higher prevalence, severity, and burden of anxiety and depression have consistently been documented in women compared to men in general, CVDs and DM populations [5, 6, 21, 22, 52]. Our study confirmed existing evidence that anxiety symptoms were more common in women than in men, irrespectively of CVDs or DM status. In contrast, the analysis of our data generally yielded marginal sex-differences in depressive symptom prevalence, except in the CVDs population in HUNT4, where men reported more depression than women. This can largely be attributed to the psychometric properties of the HADS-D subscale, also confirmed in a previous study of the HUNT2 cohort [74].

### Associations of anxiety and depression symptoms with cardiovascular diseases and diabetes

In our study, CVDs or DM was positively associated with depression and anxiety symptom risk in HUNT2 (1995–97). However, over 22 years, these associations declined, except for CVDs and symptoms of depression in men that remained across studies. Moreover, in women, DM was associated with an increased risk of psychological symptoms, greater for depression than anxiety in HUNT2 and HUNT4.

The findings that CVDs are significantly associated with symptoms of depression and anxiety are consistent with literature showing that these psychological symptoms are common after CVDs [16, 20, 75]. A literature review and meta-analysis of studies examining several vascular risk factors of late-life depression (clinically diagnosed or self-reported) found positive associations of CVDs with depression (pooled OR 1.76; 95% CI 1.52–2.04) [68]. Similarly, a meta-analysis reporting on post-stroke anxiety found self-reported anxiety in one of five stroke survivors [16]. The strength and significance of the associations of CVDs with depression and anxiety symptoms in our data changed over time across sexes, the results inconsistent compared to previous research. However, research has addressed that psychological reactions following CVDs events differ in women and men. Results from a meta-analysis of studies examining depression after CVDs diagnosis/events across sexes suggested that women experience a higher level of depression initially after a coronary heart event than men. However, in most women, symptoms tend toward improving over time, whereas men typically reported more long-lasting distress and depressive symptom burden [76].

It has been documented that several psychological conditions related to diabetes, such as stress followed by the diagnosis, feeling of the burden caused by demanding lifestyle and self-care behavior, fear of hypoglycemia, diabetes complication, and invasive procedures, may impose depression and anxiety [77]. Moreover, a meta-analysis showed that DM is associated with, on average, a 30% increased risk for both self-reported and clinically diagnosed depression [24], which partly correspond to our findings. However, these associations remained statistically significant only in women in two study waves about 20 years apart. These findings agree with a literature review on diabetes stress, an emotional state characteristical for type 2DM, that reported diabetes stress is more frequent in women than men and often followed by depression [78].

### Strengths and limitations

This study has several strengths. First, it used an internationally renowned health database to examine changes in depression and anxiety symptoms over more than 20 years in people with CVDs, DM, and adults residing in the same area without these diseases. Second, the study sample is relatively large and comprises an adult population representative of the general Norwegian adult population. Of note, HADS was specifically designed to detect anxiety and depression in patients with cardiovascular/physical conditions. Therefore, it covers core psychological symptoms of depression and anxiety, yet excludes all physical conditions (i.e., dizziness, fatigue, insomnia, and others) frequently present in both mental and physical disorders to avoid misclassification and reverse causality [13, 79].

This study also has some limitations. Depression and anxiety symptoms were based on self-rating rather than clinical interviews. This makes direct comparison with studies of diagnostic categories of anxiety and depression difficult. However, it is quite time consuming to perform diagnostic interviews, and this method is hardly feasible in large scale studies such as HUNT. In addition, using self-reported instruments with cut-off values to measure anxiety and depression levels might represent a possible source of bias, and using continuous scores could have utilized the available information along the whole range of the HADS scale. Thus, the definitive diagnosis of depression must be based on the results from the clinical interviews and the assessment of functional and somatic symptoms. However, the HADS instrument has been used in various settings, and cut-off levels have been well defined in the literature [39].

Furthermore, decreasing participation rates from HUNT2 to HUNT3 (i.e., on average, by 15.4%) may also have influenced the results. However, it should be noted that participation rates alone do not necessarily indicate selection bias [80]. Further, CVDs and DM were self-reported, which introduce the possibility that reporting bias and misclassification may have affected our results.

Overall, this study's results can mainly be generalized to middle-aged and elderly community-dwelling adults [37]. The overall prevalence rates of CVDs, DM, anxiety and depression, are likely underestimated as some individuals were too ill to participate. However, we argue that this study provides valid, up-to-date information on time trends in anxiety and depression symptoms in a nationally representative sample of adults over 22 years, across CVDs and DM status and age.

## Conclusion

We observed a declining trend in symptoms of depression and anxiety for the last two decades, irrespectively of age, sex, and CVDs or DM status. Women reported consistently more anxiety than men, whereas associations of CVDs with depression symptom remained over time in men. However, our findings indicate that depression and anxiety symptom load is still higher in people with CVDs or DM than in the general public. Anxiety and particularly depression are negatively associated with help-seeking, adherence to treatment and outcomes of CVDs and DM. Therefore, more attention to those with coexisting mental health problems during the treatment of these physical diseases should be warranted. Further research should focus on how the treatment of depression and anxiety might improve CVDs and DM outcomes, and vice versa.

## Supplementary Information

**Additional file 1.** Associations of CVDs with depression and anxiety symptoms in HUNT2 (1995–97), HUNT3 (2006–08) and HUNT4 (2017–19) at age 40, 60 and 80, multi-level logistic analysis^a^.

**Additional file 2.** Associations of DM with depression and anxiety symptoms in HUNT2 (1995–97), HUNT3 (2006–08) and HUNT4 (2017–19) at age 40, 60 and 80, multi-level logistic analysis^a.^

## Data Availability

The data used in this study are available from the HUNT databank, but restrictions apply to the availability of these data. The data were used under license for the current study and so are not publicly available. However, data are available from the authors upon reasonable request and with the included permission from the HUNT, The Regional Ethical Committee and Norwegian Data Protection Authority. The dataset used in this study, are stored in HUNT databank using a personal identification number given to all Norwegians at birth or immigration as a key identification. The HUNT Research Centre has permission from the Norwegian Data Inspectorate to store and handle these data. The HUNT data are available for scientists who wish to use them for research and non-commercial purposes, without breaching participant confidentiality. The researcher will always receive an anonymous or “de-identified” dataset after receiving approval approval of a research protocol by the Regional Ethical Committee and HUNT Research Centre. To protect participants’ privacy, HUNT Research Centre aims to limit storage of data outside HUNT databank and cannot deposit data in open repositories. HUNT databank has precise information on all data exported to different projects and are able to reproduce these on request. There are no restrictions regarding data export give approval of applications to HUNT Research. For more information about HUNT data see: https://www.ntnu.edu/hunt/data.

## Abbreviations

CI: Confidence interval
CVDs: Cardiovascular diseases
DM: Diabetes mellitus
HADS: The Hospital Anxiety and Depression Scale
HUNT: The Trøndelag Health Study
RR: Risk ratio
RD: Risk difference
