# Supplementary material for: Prevalence trends of depression and anxiety symptoms in adults with cardiovascular diseases and diabetes 1995–2019: The HUNT studies, Norway
Source: BMC Psychol. 2021 Aug 31;9:130. doi: 10.1186/s40359-021-00636-0 (PMC8406588; doi:10.1186/s40359-021-00636-0)
Supplement: Supplementary file 1 — Additional file 1. Associations of CVDs with depression and anxiety symptoms in HUNT2 (1995–97), HUNT3 (2006–08) and HUNT4 (2017–19) at age 40, 60 and 80, multi-level logistic analysisa. [file 40359_2021_636_MOESM1_ESM.docx]

| **Additional file 1.** Associations of CVDs with depression and anxiety symptoms in HUNT2 (1995-97), HUNT3 (2006-08) and HUNT4 (2017-19) at age 40, 60 and 80, multi  level logistic analysis ᵃ | | | | | | | |
| --- | --- | --- | --- | --- | --- | --- | --- |
|  |  | **HUNT2** | **HUNT3** | **HUNT4** | **HUNT2** | **HUNT3** | **HUNT4** |
|  | Age (years) | RR (95% CI) | RR (95% CI) | RR (95% CI) | RD (95% CI) | RD (95% CI) | RD (95% CI) |
| **Depression** |  |  |  |  |  |  |  |
| Women | 40 | 1.79 (1.61-1.97) | 1.61 (1.40-1.83) | 1.11 (0.93-1.28 ) | 0.07 (0.05-0.09) | 0.04 (0.03-0.06) | 0.01 (-0.00-0.02) |
|  | **60** | **1.72 (1.55-1.88)** | **1.57 (1.37-1.76)** | **1.10 (0.94-1.26)** | **0.08 (0.07-0.10)** | **0.05 (0.04-0.07)** | **0.01 (-0.01-0.03)** |
|  | 80 | 1.64 (1.50-1.79) | 1.52 (1.34-1.69) | 1.09 (0.94-1.24) | 0.10 (0.08-0.12) | 0.07 (0.04-0.09) | 0.01 (-0.01-0.03) |
|  |  |  |  |  |  |  |  |
| Men | 40 | 1.50 (1.36-1.64) | 1.27 (1.13-1.42) | 1.28 (1.13-1.43) | 0.05 (0.03-0.06) | 0.02 (0.01-0.03) | 0.02 (0.01-0.03) |
|  | **60** | **1.45 (1.33-1.57)** | **1.25 (1.12-1.38)** | **1.26 (1.12-1.39)** | **0.06 (0.04-0.07)** | **0.03 (0.01-0.04)** | **0.03 (0.01-0.04)** |
|  | 80 | 1.41 (1.30-1.52) | 1.23 (1.11-1.35) | 1.24 (1.11-1.36) | 0.07 (0.05-0.08) | 0.03 (0.02-0.05) | 0.03 (0.02-0.05) |
|  |  |  |  |  |  |  |  |
| **Anxiety** |  |  |  |  |  |  |  |
| Women | 40 | 1.36 (1.77-1.46) | 1.26 (1.14-1.38) | 1.03 (0.94-1.13) | 0.09 (0.07-0.12) | 0.05 (0.03-0.08) | 0.01 (-0.01-0.03) |
|  | **60** | **1.39 (1.28-1.49)** | **1.28 (1.15-1.40)** | **1.03 (0.93-1.14)** | **0.09 (0.07-0.11)** | **0.05 (0.03-0.07)** | **0.01 (-0.01-0.03)** |
|  | 80 | 1.43 (1.31-1.54) | 1.30 (1.16-1.44) | 1.04 (0.93-1.15) | 0.08 (0.06-0.10) | 0.04 (0.02-0.06) | 0.01 (-0.01-0.03) |
|  |  |  |  |  |  |  |  |
| Men | 40 | 1.34 (1.24-1.44) | 1.26 (1.12-1.40) | 1.06 (0.95-1.18) | 0.07 (0.05-0.09) | 0.03 (0.02-0.05) | 0.01 (-0.01-0.03) |
|  | **60** | **1.37 (1.25-1.48)** | **1.28 (1.12-1.43)** | **1.07 (0.95-1.19)** | **0.06 (0.04-0.08)** | **0.03 (0.01-0.04)** | **0.01 (-0.01-0.03)** |
|  | 80 | 1.44 (1.30-1.58) | 1.32 (1.14-1.50) | 1.08 (0.94-1.22) | 0.04 (0.03-0.05) | 0.02 (0.01-0.03) | 0.01 (-0.00-0.02) |
| **Abbreviations**: CVDs, Cardiovascular diseases; HUNT, The Trøndelag Health Study; RR, Risk ratio; RD, Risk difference; CI, Confidence Interval.  ᵃAdjusted for age and age squared. Risk Ratio (RR) and Risk Difference (RD) between individuals reporting CVDs and no-CVDs (ref.) at age 40, 60 and 80 | | | | | | | |
