# Supplementary material for: Prevalence trends of depression and anxiety symptoms in adults with cardiovascular diseases and diabetes 1995–2019: The HUNT studies, Norway
Source: BMC Psychol. 2021 Aug 31;9:130. doi: 10.1186/s40359-021-00636-0 (PMC8406588; doi:10.1186/s40359-021-00636-0)
Supplement: Supplementary file 2 — Additional file 2. Associations of DM with depression and anxiety symptoms in HUNT2 (1995–97), HUNT3 (2006–08) and HUNT4 (2017–19) at age 40, 60 and 80, multi-level logistic analysisa. [file 40359_2021_636_MOESM2_ESM.docx]

| **Additional file 2.** Associations of DM with depression and anxiety symptoms in HUNT2 (1995-97), HUNT3 (2006-08) and HUNT4 (2017-19) at age 40, 60 and 80, multi-level  logistic analysis ᵃ | | | | | | | |
| --- | --- | --- | --- | --- | --- | --- | --- |
|  |  | HUNT2 | HUNT3 | HUNT4 | HUNT2 | HUNT3 | HUNT4 |
|  | Age (years) | RR (95% CI) | RR (95% CI) | RR (95% CI) | RD (95% CI) | RD (95% CI) | RD (95% CI) |
| **Depression** |  |  |  |  |  |  |  |
| Women | 40 | 1.40 (1.18-1.62) | 1.20 (0.98-1.42) | 1.26 (1.06-1.45) | 0.04 (0.02-0.06) | 0.01 (-0.00-0.03) | 0.02 (0.00-0.03) |
|  | **60** | **1.36 (1.17-1.56)** | **1.18 (0.98-1.38)** | **1.24 (1.06-1.42)** | **0.04 (0.02-0.07)** | **0.02 (-0.00-0.04)** | **0.02 (0.01-0.04)** |
|  | 80 | 1.32 (1.15-1.50) | 1.17 (0.99-1.35) | 1.22 (1.06-1.38) | 0.05 (0.03-0.08) | 0.02 (-0.00-0.05) | 0.03 (0.01-0.05) |
|  |  |  |  |  |  |  |  |
| Men | 40 | 1.24 (1.04-1.44) | 1.08 (0-89-1.77) | 1.09 (0.93-1.26) | 0.02 (0.00-0.04) | 0.01 (-0.01-0.02) | 0.01 (-0.01-0.02) |
|  | **60** | **1.22 (1.04-1.40)** | **1.07 (0.90-1.24)** | **1.09(0.94-1.23)** | **0.03 (0.01-0.05)** | **0.01 (-0.01-0.03)** | **0.01 (-0.01-0.03)** |
|  | 80 | 1.20 (1.04-1.35) | 1.07 (0.91-1.22) | 1.08 (0.94-1.21) | 0.03 (0.01-0.06) | 0.01 (-0.01-0.03) | 0.01 (-0.01-0.03) |
|  |  |  |  |  |  |  |  |
| **Anxiety** |  |  |  |  |  |  |  |
| Women | 40 | 1.12 (1.00-1.24) | 1.05 (0.92-1.18) | 1.13 (1.02-1.23) | 0.03 (-0.00-0.06) | 0.01 (-0.02-0.04) | 0.03 (0.01-0.05) |
|  | **60** | **1.12 (1.00-1.25)** | **1.05 (0.91-1.19)** | **1.13 (1.02-1.24)** | **0.03 (-0.00-0.06)** | **0.01 (-0.02-0.03)** | **0.03 (0.01-0.05)** |
|  | 80 | 1.13 (0.99-1.27) | 1.05 (0.91-1.20) | 1.14 (1.02-1.26) | 0.03 (-0.00-0.05) | 0.01 (-0.01-0.03) | 0.03 (0.00-0.05) |
|  |  |  |  |  |  |  |  |
| Men | 40 | 1.20 (1.04-1.35) | 1.12 (0.94-1.31) | 1.11 (0.98-1.24) | 0.04 (0.01-0.07) | 0.02 (-0.01-0.04) | 0.02 (-0.00-0.04) |
|  | **60** | **1.21 (1.04-1.38)** | **1.13 (0.93-1.33)** | **1.12 (0.97-1.26)** | **0.03 (0.01-0.06)** | **0.01 (-0.01-0.03)** | **0.02 (-0.00-0.04)** |
|  | 80 | 1.24 (1.05-1.44) | 1.15 (0.92-1.37) | 1.13 (0.97-1.30) | 0.03 (0.01-0.05) | 0.01 (-0.00-0.02) | 0.01 (-0.00-0.03) |
| **Abbreviations**: DM, Diabetes mellitus; HUNT, The Trøndelag Health Study; RR, Risk difference; RD, Risk Ratio; CI, Confidence Interval.  ᵃAdjusted for age and age squared. Risk Ratio (RR) and Risk Difference (RD) between individuals reporting DM and no-DM (ref.) at age 40, 60 and 80 | | | | | | | |
